# Reviewer Acknowledgement

**DOI:** 10.1002/rmb2.12555

**Published:** 2024-01-20

The publication of invaluable papers in the Reproductive Medicine and Biology depends on the prompt careful review of submitted manuscripts. We thank the Editorial Board members, the Editorial Advisory Board members, and the following experts for reviewing manuscripts from November 1, 2022 to October 31, 2023.

Kristian Almstrup

Hisao Ando

Yoshimasa Asada

Yoshiko Asai

Atsushi Azumaguchi

Fumihisa Chishima

Ludovic Dumont

Noritoshi Enatsu

Yuji Endo

Kenji Ezoe

Shunsaku Fujii

Masakatsu Fujinoki

Maki Fukami

Aisaku Fukuda

Atsushi Fukui

Hiroaki Funahashi

Tatsuro Furui

Masataka Furuya

Jun Hagiuda

Toshio Hamatani

Kenshiro Hara

Miyuki Harada

Tomonobu Hasegawa

Yuko Hasegawa

Yasushi Hirota

Kentaro Ichioka

Masashi Iijima

Masahito Ikawa

Tomomoto Ishikawa

Richard Ivell

Toshiyuki Iwahata

Takeshi Iwasa

Takuya Iwasawa

Shoichiro Iwatsuki

Hiroki Izumi

Rajasingam Jeyendran

Seung Chik Jwa

Daisuke Kadogami

Takeshi Kajihara

Naomi Kashiwazaki

Mayuko Kato

Kiyotaka Kawai

Yasushi Kawano

Khaleque Khan

Kazuhiro Kikuchi

Fuminori Kimura

Naoko Kimura

Hiroshi Kishi

Satoshi Kishigami

Yoshikazu Kitahara

Michio Kitajima

Masato Kobanawa

Hideyuki Kobayashi

Hiroshi Kobayashi

Ko Kobayashi

Kouji Komatsu

Akira Komiya

Tomomi Kotani

Koji Kugu

Sohei Kuribayashi

Keiji Kuroda

Yoshimitsu Kuwabara

Tomoichiro Kuwazuru

Eri Maeda

Ryo Maekawa

Hirotaka Masuda

Hidehiko Matsubayashi

Mitsunori Matsuo

Shinya Matsuzaki

Yasuyuki Mio

Hidehiko Miyake

Satoshi Mizuno

Adel R Moawad

Taisuke Mori

Tetsunori Mukaida

Koji Nakagawa

Tomoko Nakamura

Tatsuya Nakano

Yoshiharu Nakaoka

Ayako Nishimoto‐Kakiuchi

Miho Ohsugi

Hidetaka Okada

Keisuke Okada

Osamu Okitsu

Masanori Ono

Aki Oride

Satoko Osuka

Hyun Jun Park

Toshihiro Sakurai

Osamu Samura

Masahide Shiotani

Masato Shirai

Yodo Sugishita

Seido Takae

Toshifumi Takahashi

Yasushi Takai

Akie Takebayashi

Teppei Takeshima

Hiroki Takeuchi

Kentaro Takezawa

Isao Tamura

Satoshi Tanaka

Fuminori Taniguchi

Hisanori Taniguchi

Yuka Taniguchi

Hidetaka Tasaki

Hiroyuki Tomari

Kazuhisa Tomita

Isao Tsuji

Akira Tsujimura

Hiroshi Uchida

Yoshihisa Uenoyama

Takashi Umehara

Osamu Wada‐Hiraike

Takuya Wakai

Tomohiko Wakayama

Mitsutoshi Yamada

Yuri Yamamoto

Yasuhisa Yamashita

Kaoru Yanagida

Tsukasa Yoshida

Osamu Yoshino

